# Assessment of the correlation of the tear breakup time with quality of vision and dry eye symptoms after SMILE surgery

**DOI:** 10.1007/s10792-021-02086-4

**Published:** 2021-10-28

**Authors:** Christoph Palme, Fiona Mulrine, Richard N. McNeely, Bernhard Steger, Shehzad A. Naroo, Jonathan E. Moore

**Affiliations:** 1 Cathedral Eye Clinic, 89-91 Academy Street, Belfast, BT1 2 LS Northern Ireland UK; 2 Department of Ophthalmology, Medical University of Innsbruck, Innsbruck, Austria; 3 College of Health and Life Sciences, Aston University, Birmingham, UK; 4 Tianjin Medical University, Tianjin, China

**Keywords:** Fluorescein tear film breakup time (FTBUT), Small incision lenticule extraction (SMILE), Dry eye, Quality of vision (QOV), Ocular surface disease index (OSDI)

## Abstract

**Purpose:**

It is well reported that dry eye symptoms can increase after many refractive surgery procedures. This study aims to provide a clinical understanding of the correlation of fluorescein tear film breakup time (FTBUT) with quality of vision (QoV) and dry eye symptoms following small incision lenticule extraction surgery (SMILE).

**Methods:**

Patients electing to have SMILE surgery were subdivided into 2 groups: Group 1 included short preoperative FTBUTs of 3 to 6 seconds (s); Group 2 included long FTBUTs of ≥ 8 s. Uncorrected distance visual acuity, corrected distance visual acuity, manifest refraction, FTBUT, QoV and Ocular Surface Disease Index (OSDI) questionnaires were recorded 1 and 6 months postoperatively.

**Results:**

Thirty-nine subjects were included in each group. There was no significant difference in visual outcomes between the 2 groups at both the 1- and 6-month postoperative assessments. FTBUT remained significantly lower in group 1. Oxford staining was initially higher for group 1 at 1 month (*P* = 0.007), but there was no significant difference at 6 months (*P* = 0.180). There was no significant difference in QoV or OSDI scores between the 2 groups at both postoperative visits.

**Conclusions:**

Low preoperative FTBUT (3–6 s) does not appear to negatively affect postoperative visual outcomes or results in a greater likelihood of dry eye symptoms and poor ocular surface compared to eyes with a longer preoperative FTBUT. These results suggest that a low preoperative FTBUT does not necessarily increase the likelihood of poor visual acuity, dry eyes symptoms, or poor ocular surface outcomes following SMILE surgery.

## Introduction

Dry eye disease (DED) is a well-known complication of laser-assisted in situ keratomileusis (LASIK) post-treatment of refractive errors. While the majority of all LASIK patients may suffer from some degree of dry eye symptoms within 1 month of surgery as part of the healing response [1], these symptoms can remain in up to 36% of patients 6-month postoperatively [2] leading to decreased patient satisfaction and persistent discomfort [3]. Mechanical and postoperative inflammatory factors have been identified in the multifactorial driven pathophysiology [4]. Creation of the LASIK flap within the anterior corneal stroma as well as stromal ablation severs most of the sensory corneal nerves and thus disrupts the neural feedback loop to ocular surface and lacrimal gland and the blink mechanism, which is important in maintaining a healthy ocular surface [4–6]. Consequently, corneal sub-basal nerve density is significantly decreased after LASIK and it may take up to 5 years for recovery [7].

The introduction of small incision lenticule extraction (SMILE) has enabled a corneal laser refractive treatment which is less invasive resulting in reduced impact upon both the corneal biomechanics and tear film. The small corneal surface incision and the formation of a refractive lenticule by femtosecond laser deeper within the posterior corneal stroma result in preservation of more anterior corneal nerve fibers and improve the postoperative health of the ocular surface [8]. A history of preoperative dry eye has been identified as one of the main risk factors of experiencing dry eye symptoms after LASIK [9]. However, with the less invasive impact of SMILE on the cornea and, therefore, on DED, more patients may be safely treated despite their preexisting condition. Especially as more patients with contact lens intolerance or DED are seeking independence from glasses, SMILE may offer a more suitable treatment. Furthermore, various types of laser corneal refractive surgery including SMILE surgery have been associated with increased ocular aberration measurements [10]. Altered tear films demonstrating short FTBUTs have similarly been associated with ocular aberrations [11]. These findings raise the question, as to whether short FTBUTs may influence the perceived quality of vision (QoV) of SMILE patients after surgery. A FTBUT of 10 seconds (s) or greater is still deemed as normal [12]; however, it is well accepted now that a high percentage of laser refractive patients are treated safely with a FTBUT well under 10 s. Therefore, this retrospective study sought to assess the correlation of preoperative FTBUT with postoperative QoV and dry eye symptoms following SMILE surgery.

## Methods

This retrospective non-randomized study included 100 consecutive patients (200 eyes) undergoing bilateral SMILE surgery for their refractive error between January 2017 and January 2018. Patients were divided into two groups based upon their preoperative FTBUT. According to a previous study, patients experienced mild dry eye syndrome with a FTBUT of 7 s and below [13]. In this cohort of 200 eyes, the mean FTBUT was 7.5 ± 2.4 s. The mean FTBUT of each patient’s two eyes was recorded and patients were then subdivided based upon this mean score. Since the study was aiming to investigate the difference between two distinct groups, any patient with a mean FTBUT of 7 s was excluded (*n* = 22). Patients with a mean FTBUT of 3 to 6 s were categorized as low FTBUT and labelled Group 1 (*n* = 39). The lowest FTBUT was 3 s, as patients with significant dry eye signs were considered unsuitable for laser vision correction. Patients with a mean FTBUT of ≥ 8 s were categorized into the long FTBUT and labelled Group 2 (*n* = 39). Furthermore, the monocular FTBUT was compared between each patients’ two eyes to determine if a patient had a FTBUT in group 1 and their fellow eye was in group 2. This was not found to be the case with any patient in this study.

Exclusion criteria were as follows:

- Refractive stability less than 2 years.
- Uncontrolled dry eye disease.
- History of glaucoma.
- Past retinal detachment.
- Ocular inflammation.
- Corneal surgery or disease.
- Neuro-ophthalmic disease.
- Macular disease.

### Patient assessment

Full ophthalmic assessment was performed on all patients preoperatively. The examination included a medical history, uncorrected (UDVA) and corrected (CDVA) distance visual acuities, autorefraction (OPD-Scan II ARK-10000, Nidek Co., Gamagori, Japan), subjective and manifest refraction (RT-5100 Auto Phoropter Head, Nidek Co., Gamagori, Japan), keratometry, topography, slit-lamp examination, Goldmann tonometry, dilated fundus examination and retinal optical coherence tomography (Cirrus 4000 OCT; Carl Zeiss Meditec, Jena, Germany). Visual acuity measures were evaluated with logMAR distance charts at 6 m.

FTBUT was measured preoperatively and postoperatively. The patient was asked to blink a couple of times to allow uniform distribution. Once achieved, the FTBUT was measured, with a stopwatch, as the interval between the last blink and the first appearance of a dry spot on the corneal surface using a broad beam of cobalt blue light with a Wratten filter to aid viewing. The test was repeated three times consecutively at each visit and an average was taken [12–14]. FTBUT was measured following completion of visual acuity testing and scanning.

Patients were examined at 1 month and at 6 months postoperatively. Full ophthalmic examination was performed postoperatively using the same techniques as preoperatively.

A specifically developed QoV questionnaire was completed postoperatively [15]. The questionnaire assessed the severity of symptoms that patients experienced. Patients responded on a Likert scale either not at all (0), a little (1), quite (2) or very (3). Additionally, patients were asked regarding their own subjective view of their total QoV on a linear scale of 0 to 10 (0 the worst, 10 the best) to gain a better understanding of each patient’s postoperative satisfaction. Standard categorical analysis techniques were utilized to look for statistical differences between each item between groups.

To assess dry eye symptoms, the ocular surface disease index (OSDI) was used, which was introduced in 1997 by the Outcomes Research Group (Allergan Inc., Irvine, CA, USA) [16]. It consists of 12 questions graded from a scale of 0 to 4, where 0 indicates none of the time; 1, some of the time; 2, half of the time; 3, most of the time; and 4, all of the time. The total OSDI score was then calculated by the following formula with a score of 100 as the highest, with higher scores representing more disability: OSDI = [(sum of scores for all questions answered) × 100]/[(total number of questions answered) × 4] [16, 17]. The OSDI scores can be used to define ocular surface disability due to dry eye disease and are grouped as normal/no disability: 0–12, mild: 13–22, moderate: 23–32, or severe: 33–100 [18].

Each patient gave their informed consent for the surgical procedure and an audit of the findings for this study, including for publication. The Tenets of the Declaration of Helsinki were adhered to throughout this work.

### Surgical technique

All SMILE surgeries were performed by the same experienced surgeon (JEM) using topical anesthesia (0.4% oxybuprocaine hydrochloride). Bilateral SMILE was performed using a femtosecond laser (Visumax, Carl Zeiss Meditec, Jena, Germany) with a 135 µm cap, 6.5-mm optical zone, and a 2-o’clock small tunnel incision. After careful separation of the lenticule, it is extracted through the corneal incision with a blunt spatula. After surgery, the patient was assessed on a slit lamp and one drop of fluorescein dye eye drops applied in both eyes. Postoperative therapy includes the combination of tobramycin 0.3% and dexamethasone 0.1% as well as ofloxacin 0.3% eye drops twice daily for one week. Patients were encouraged to use lubricating drops as required postoperatively and were encouraged to use them often in addition to the routine suggested by the clinician. Use of lubricating drops was therefore not recorded as maximal optimization of the ocular surface was enforced in all patients and subsequently adapted to the clinical need of the patient.

### Statistical analysis

Statistical analysis was performed using SPSS for Windows (Statistical Package for the Social Sciences, Version 22, Chicago, Illinois, USA) and Excel (Microsoft; Redmond, Washington, USA). The Independent Samples *t* test was used for parametric analysis. When assessing nonparametric data, a Mann–Whitney U test was utilized. Following the methods outlined by Goodall et al. [19], calculations indicated that for this study to have 80% statistical power, the sample size required was more than 36 patients per group. A standard deviation of 0.90 for the QoV score was used and a difference of 0.6 in QoV was considered to be clinically significant. For all statistical analysis, the level of significance was *P* < 0.05.

## Results

### Demographics

Group 1 consisted of 39 patients (FTBUT 3 to 6 s) and Group 2 consisted of 39 patients (FTBUT ≥ 8 s). The mean age in group 1 was 32 ± 5.7 years compared to 29 ± 4.4 years in group 2. There was a higher percentage of females in group 1, with 79% compared to 38% in group 2. A statistically significant difference in FTBUT between the groups (*P* < 0.001, independent *t* test) is displayed. There was no statistical difference in preoperative visual and refractive parameters. The preoperative QoV score was 8.5 ± 1.3 for the night and 9.0 ± 1.2 for day in Group 1, and Group 2 showed a night rating of 8.40 ± 1.8 and a day rating of 8.8 ± 1.8. There was no statistically significant difference between the groups for the day (*P* = 0.63, independent *t* test) or nighttime QoV scores (*P* = 0.71, independent *t* test). The OSDI scores are within the normal range in both groups, and there is no significant difference in OSDI between groups 1 and 2 (*P* = 0.52, independent *t* test). Particularly for group 1, the OSDI score is 1.2 ± 1.9, which suggests there is no symptomatic disability due to ocular surface dryness, despite a short FTBUT.

### Quality of vision (QoV) and photopic phenomena

Table 1 compares and outlines the individual symptom responses and the overall QoV scores 1 and 6 months after SMILE surgery. No significant differences in the overall day and night QoV scores at both 1 and 6 months were found. Glare was the only symptom that was found to be statistically different between the 2 groups at 1 month (*P* = 0.011, Wilcoxon Signed Rank); however, there was no statistically significant difference at 6 months (*P* = 0.059, Wilcoxon Signed Rank).

**Table 1:** Between-group comparison of QOV at 1 and 6 months postoperatively

1 month	Group 1	Group 2	*P* value
QOV night	8.97 ± 1.24	9.26 ± 0.78	0.947
QOV day	9.03 ± 0.95	9.20 ± 0.86	0.571
How much does glare bother you?	0.64 ± 0.81	0.00 ± 0.00	0.011
How much do the haloes bother you?	0.50 ± 0.50	0.00 ± 0.00	0.157
How much do the starbursts bother you?	0.43 ± 0.50	0.10 ± 0.30	0.655
How much does blurred vision bother you?	0.38 ± 0.70	0.01 ± 0.26	0.564
How much do fluctuations bother you?	0.33 ± 0.67	0.19 ± 0.39	1.00

6 month	Group 1	Group 2	*P* value
QOV night	9.06 ± 1.25	9.26 ± 0.95	0.658
QOV day	9.33 ± 0.91	9.62 ± 0.60	0.681
How much does glare bother you?	0.19 ± 0.58	0.00 ± 0.00	0.059
How much do the haloes bother you?	0.00 ± 0.00	0.00 ± 0.00	0.317
How much do the starbursts bother you?	0.00 ± 0.00	0.00 ± 0	0.564
How much does blurred vision bother you?	0.15 ± 0.44	0.00 ± 0.37	0.589
How much do fluctuations bother you?	0.06 ± 0.24	0.09 ± 0.28	1.00

### Visual and refractive outcomes

A statistical difference mean spherical equivalent (MSE) between the 2 groups at 1 month (*P* = 0.003, independent *t* test) and 6 months (*P* = 0.001, independent *t* test) was found. There was no statistically significant difference in CDVA at 1 month (*P* = 0.460, independent *t* test) and 6 months (*P* = 0.795, independent *t* test). A statistically significant difference in refractive sphere between the 2 groups at 1 month (*P* = 0.001, independent *t* test) and at 6 months (*P* = 0.002, independent *t* test) was found.

### OSDI and dry eye clinical findings

**Fig. 1:**
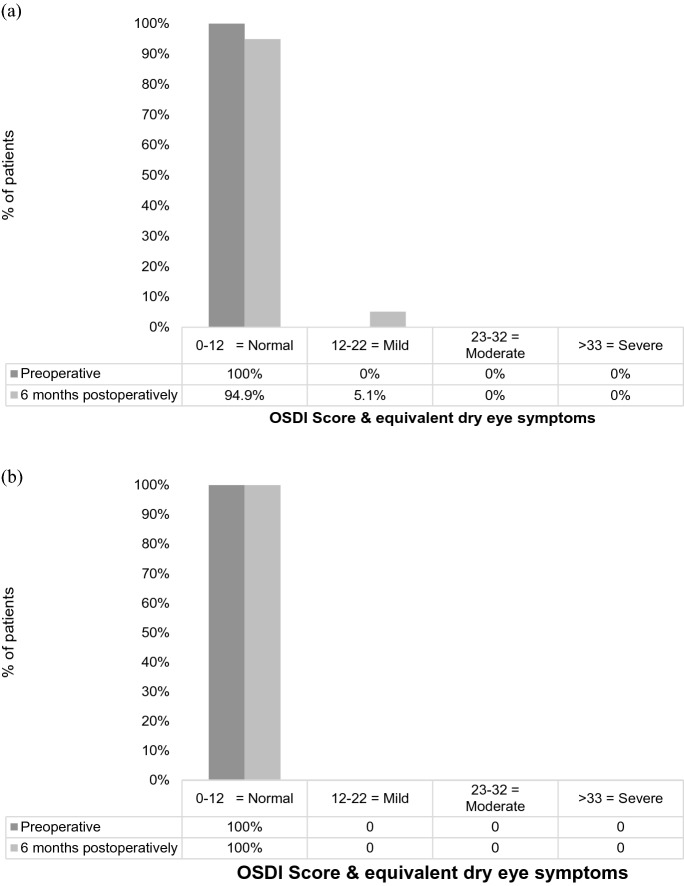
Percentage of patients reporting dry eye symptoms based on ocular surface disease index (OSDI) preoperatively and 6 months after surgery. **a** Group 1, **b** Group 2

Group 1 showed a significantly higher Oxford staining (*P* = 0.007, Mann–Whitney *U*) at 1 month, but no significant difference was evident between the 2 groups at 6 months. With a FTBUT of 5.15 ± 1.7 s at 1 month and 6.79 ± 2.9 at 6 month, Group 1 retained a lower FTBUT compared to Group 2 with a FTBUT of 5.63 ± 1.97 at 1 month (*P* = 0.001, independent *t* test) and of 7.31 ± 2.1 (*P* < 0.001, independent *t* test) at 6 month over the whole observation period. There was no significant difference in postoperative OSDI scores between the 2 groups. Figure 1 displays the percentage of patients within the graded scales of the OSDI questionnaire and how this altered over the two postoperative periods. All 39 patients in each group reported ‘normal’ OSDI scores preoperatively and 37 and 39 patients in the two respective groups at 6 months. Two patients reported ‘mild’ OSDI scores at 6 months in group 1.

## Discussion

It is widely understood that DED is a well-known complication of LASIK and is a cause of reduced satisfaction following refractive surgery [3]. This study aimed to assess if the SMILE procedure results in a reduced impact on the ocular surface, indicating that it is a safe procedure even in the presence of mild dry eye. The tear film impacts the clarity of an image and has significant visual importance; therefore, in this study, the FTBUT was considered as the defining preoperative parameter. Furthermore, it is relatively quick and simple to assess within the normal clinical routine and is a widely utilized and is an instantly recognizable clinical measurement. However, there is reported variability with this measurement [17]. An earlier study, by the authors, reported that dry eye symptoms significantly correlate to a FTBUT of ≤ 7 s [9]. Therefore, in choosing to exclude the mean FTBUT of 7 s in this study, it served as a qualitative marker to clearly distinguish between the preoperative FTBUT groups. This decision is further supported by Lee et al., who reported higher reproducibility for FTBUT ≤ 5 s in dry eyes than for normal eyes thus indicating the reliability of a short FTBUT [20]. This gave two distinct groups, one group had a low preoperative FTBUT (3–6 s) and the other a high preoperative FTBUT (≥ 8 s). The groups were based upon the mean of the two eyes with the main outcome measurement of this study being subjective outcomes which is a binocular assessment. Assessment of the monocular FTBUT of each patient showed that no patients had one eye that would be defined by this study as having a low FTBUT and the fellow eye a long FTBUT. Furthermore, it was found that if the lowest FTBUT of each patient’s two eyes was used it would not have changed the patients in each group in this study and therefore the mean FTBUT was utilized.

This study assessed the correlation of preoperative FTBUT with both the objective visual and refractive outcomes and the subjective QoV and OSDI in patients who underwent SMILE surgery.

FTBUT was the defining parameter preoperatively and it was found that the mean FTBUT for group 1 remained significantly lower than group 2 at both the 1-month and 6-month postoperative assessments. Studies have shown that a lower FTBUT impacts the optical image on the retina [21, 22]. However, in this current study, there was no significant difference between objective visual and refractive outcomes. Both groups showed excellent UDVA at both postoperative assessments, with no significant difference. There was a significant difference in MSE; however, it appears that this difference is not clinically significant. The UDVA achieved in this current study is excellent and appears to be superior to that found in other studies [23]. Similar to the objective visual outcomes, there is no significant difference between the two groups in subjective reports of blurred vision or fluctuation of vision. A low preoperatively FTBUT appears to cause more glare at 1 month; however, this reduced at 6 months. There appears to be a high level of postoperative satisfaction in both groups with 9.03 ± 0.95 and 9.20 ± 0.86 at 1 month and 9.33 ± 0.91 and 9.62 ± 0.60 at 6 months in the two respective groups. It appears that the lower FTBUT group does not significantly report worse day or nighttime QoV scores postoperatively (Table 1).

It has been found previously that corneal staining following SMILE is significantly less when compared to LASIK [24]. Comparison of the corneal staining in this current study showed no significant difference preoperatively between the two groups. Patients are not recommended to proceed with surgery if significant corneal staining is present and are advised to optimize their tear film and will be assessed in the clinic again prior to proceeding with any surgery. Postoperatively at the 1 month assessment, group 1 showed significantly more corneal staining; however, the Oxford score remained low at 0.32 ± 0.59. There was no significant difference between the two groups at 6 months. Similarly, there was no significant difference between groups in conjunctival folds at either postoperative assessment. The implication of SMILE has been reported to have a less severe impact upon the corneal innervation and thus the anterior surface [25], which appears to concur with this current study. It appears that having a lower preoperative FTBUT prior to SMILE surgery does not significantly increase the presence of clinical signs of dry eye postoperatively. This is also reflected in postoperative subjective outcomes through the OSDI questionnaire. This study highlighted a low level of OSDI, and similarly to the QoV outcomes, there was no significant difference between the two groups. Figure 1 displays the number of patients within the defined severity grades within the OSDI questionnaire. In group 1, all patients reported “normal” symptoms preoperatively, however 6 months postoperatively 2 patients reported to have “mild” dry eye symptoms. This further suggests that having a lower FTBUT does not significantly impact a patient’s subjective dry eye symptoms.

This study appears to suggest that the presence of a low FTBUT preoperatively is not a contraindication to proceeding with SMILE surgery. Patients with a lower FTBUT preoperatively (3 to 6 s) do not report significantly lower QOV or worse dry eye symptoms. This is also reflected in the objective visual outcomes and clinical signs of dry eye. This study appears to highlight that SMILE surgery is safe to perform in patients with a low FTBUT. This is supported by a previous which suggests that SMILE surgery can result in a decrease in dry eye symptoms postoperatively [26].

The limitations of this study include the heterogenous groups reported. There was a significant difference in age between the two groups, with the mean age and standard deviation 32 ± 5.7 in group 1, and 29 ± 4.4 in group 2. The gender between the two groups may also have had an impact upon the outcomes; therefore, these factors will be assessed in future studies. However, the impact of postoperative lubricating efforts is not reported by this study and may confound the results. Usually patients, who are more aware of their symptoms tend to use their drops more regularly. This should however decrease the difference between both groups in this study and diminish the effect found. Therefore, it could be concluded that this study depicts significant clinical results despite its limitations, which may help for preoperative selection of patients for refractive surgery.

With the recent publication of the “Refractive Errors & Refractive Surgery Preferred Practice Pattern” by the American Academy of Ophthalmology, uncontrolled dry eye syndrome is mentioned as contraindication for corneal refractive surgery [27]. Toda et al. found that the efficacy and safety of LASIK were not affected by preexisting dry eye status, but resulted in more severe postoperative dry eye [28]. Similarly, this paper highlights that the preoperative tear film does not significantly impact the postoperative QoV following SMILE surgery. Therefore, while being eligible for laser refractive surgery, well-controlled DED with low preoperative FTBUT will benefit from additional management and assessment before and after surgery.

In conclusion, this study has demonstrated that SMILE surgery would appear subjectively and objectively safe to carryout despite low preoperative FTBUTs. However, as this study has concentrated on the impact of the dry eye indicator FTBUT only, further studies are necessary to investigate the impact of different dry eye measures to identify eligible patients for refractive surgery. Especially, with the recent development of SMILE and of additional therapeutic options for DED, refractive surgery may become available for more patients.

## Data Availability

Available upon reasonable request.
